# Natural Killer T Cells Are Involved in Atherosclerotic Plaque Instability in Apolipoprotein-E Knockout Mice

**DOI:** 10.3390/ijms222212451

**Published:** 2021-11-18

**Authors:** Yoshinori Ohmura, Naoki Ishimori, Akimichi Saito, Takashi Yokota, Shunpei Horii, Satoshi Tokuhara, Kazuya Iwabuchi, Hiroyuki Tsutsui

**Affiliations:** 1 Department of Cardiovascular Medicine, Hokkaido University Graduate School of Medicine, Kita-15 Nishi-7, Kita-ku, Sapporo 060-8638, Japan; Yoshi-2@med.hokudai.ac.jp (Y.O.); akimi@med.hokudai.ac.jp (A.S.); t-yokota@med.hokudai.ac.jp (T.Y.); tokuhara@med.hokudai.ac.jp (S.T.); 2 Department of Cardiovascular Medicine, National Defense Medical College, Namiki 3-2, Tokorozawa 359-0042, Japan; shumhorii@outlook.jp; 3 Department of Immunology, Kitasato University School of Medicine, Kitasato 1-15-1, Minami-ku, Sagamihara 252-0374, Japan; akimari@kitasato-u.ac.jp; 4 Department of Cardiovascular Medicine, Kyushu University Graduate School of Medicine, Maidashi 3-1-1, Higashi-ku, Fukuoka 812-8582, Japan; htsutsui@cardiol.med.kyushu-u.ac.jp

**Keywords:** α-galactosylceramide, apolipoprotein E knockout mice, atherosclerosis, brachiocephalic artery, macrophages, matrix metalloproteinase, natural killer T cells, plaque instability

## Abstract

The infiltration and activation of macrophages as well as lymphocytes within atherosclerotic lesion contribute to the pathogenesis of plaque rupture. We have demonstrated that invariant natural killer T (iNKT) cells, a unique subset of T lymphocytes that recognize glycolipid antigens, play a crucial role in atherogenesis. However, it remained unclear whether iNKT cells are also involved in plaque instability. Apolipoprotein E (apoE) knockout mice were fed a standard diet (SD) or a high-fat diet (HFD) for 8 weeks. Moreover, the SD- and the HFD-fed mice were divided into two groups according to the intraperitoneal injection of α-galactosylceramide (αGC) that specifically activates iNKT cells or phosphate-buffered saline alone (PBS). ApoE/Jα18 double knockout mice, which lack iNKT cells, were also fed an SD or HFD. Plaque instability was assessed at the brachiocephalic artery by the histological analysis. In the HFD group, αGC significantly enhanced iNKT cell infiltration and exacerbated atherosclerotic plaque instability, whereas the depletion of iNKT cells attenuated plaque instability compared to PBS-treated mice. Real-time PCR analyses in the aortic tissues showed that αGC administration significantly increased expressional levels of inflammatory genes such as IFN-γ and MMP-2, while the depletion of iNKT cells attenuated these expression levels compared to those in the PBS-treated mice. Our findings suggested that iNKT cells are involved in the exacerbation of plaque instability via the activation of inflammatory cells and upregulation of MMP-2 in the vascular tissues.

## 1. Introduction

Clinical complications of atherosclerosis, such as myocardial infarction and ischemic stroke, result from the sudden thrombotic occlusion of the artery that arises from atherosclerotic plaques not necessarily causing flow-limiting stenoses [1]. Physical disruption of the atherosclerotic plaque is attributable to rupture of the fibrous cap that overlies the lipid core with the plaque [2]. Interstitial collagen fibers normally confer the structural stability of the fibrous cap on the plaque. Atherosclerosis results from complex inflammatory processes between hematocytes and vascular tissues [3]. In the early stage of atherosclerosis (characterized by fatty-streak lesions), macrophages and T lymphocytes are frequently found in the atherosclerotic lesions, whereas, in the late stage after progression of atherosclerosis, aggregation of activated macrophages, T lymphocytes, and smooth muscle cells (SMC) is associated with the development of complex atherosclerotic lesions. It has been reported that T lymphocyte activation markedly increases production of interferon (IFN)-γ and strongly inhibits the synthesis of collagens by vascular SMC. IFN-γ also inhibits the proliferation of vascular SMCs, leading to instability of the plaque due to reduction of collagen-synthesizing cellular component in the plaque. Furthermore, it has been demonstrated that T lymphocytes in atherosclerotic plaques activate macrophages via increased expression of matrix metalloproteinase (MMP)-2 and MMP-9. Accordingly, T cells play an important role in regulation of SMCs and macrophages, both of which may restore the integrity of the fibrous cap of the plaque and finally prevent plaque rupture.

Natural killer T (NKT) cells are innate-like T lymphocytes that share surface receptors with both conventional T lymphocytes (TCR; T cell receptors) and natural killer (NK) cells (NK1.1). These NKT cells recognize glycolipid antigens presented by the major histocompatibility complex (MHC) class I-like molecule CD1d. Upon activation NKT cells rapidly and robustly produce a mixture of T helper type 1 (Th1) and Th2 cytokines such as IFN-γ and interleukin (IL)-4 that shape subsequent adaptive immune responses on activation [4]. Thus, NKT cells can function as a bridge between the innate and adaptive immune systems, and orchestrate tissue inflammation. Indeed, we have demonstrated that invariant NKT (iNKT) cells, which are the major subset of NKT cells and possess a restricted TCR expression (Vα14-Jα18 in mice and Vα24-Jα18 in humans), are involved in atherogenesis, and their activation decreased collagen content and increased cellularity within the atherosclerotic lesions in apolipoprotein E (apoE) knockout mice [5]. The administration of α-galactosylceramide (αGC), a specific activator for iNKT cells [6], to apoE knockout mice decreased the collagen content in the aortic atherosclerotic lesions stained with Elastica-Masson [5]. We also reported that the prevalence of iNKT cells in peripheral blood was significantly decreased in patients with unstable angina compared to control subjects [7]. These findings suggest that iNKT cell’s activation may amplify the local inflammatory response and be involved in the pathophysiology of plaque instability leading to plaque rupture. However, the role of iNKT cells in plaque instability is completely unknown.

An animal model of spontaneously occurred plaque rupture was proposed by Johnson et al., in which ruptured atherosclerotic plaques occurred in the brachiocephalic artery from male apoE knockout mice after 8 weeks of high-fat diet (HFD) feeding [8,9]. In the present study, we examined whether iNKT cells were involved in the stability of atherosclerotic plaques and the inflammation of aortic tissues in this model of plaque rupture following the iNKT cell’s activation after administration of αGC. In addition, we examined the effects of iNKT cell’s depletion on this disease process using apoE/Jα18 double knockout mice fed an HFD. We herein report that activation of iNKT cells plays a key role in instability of the atherosclerotic plaques.

## 2. Results

### 2.1. Animal Characteristics

ApoE knockout mice were fed a standard diet (SD) or HFD for 8 weeks and the SD- and the HFD-fed mice were further divided into two groups according to the intraperitoneal injection of αGC (SD-αGC and HFD-αGC) or phosphate-buffered saline (PBS; SD-PBS and HFD-PBS) twice a week for 8 weeks. ApoE/Jα18 double knockout mice were also fed an SD or HFD for 8 weeks (SD-KO and HFD-KO).

HFD feeding did not affect the body weight, serum high-density lipoprotein (HDL)-cholesterol, free fatty acid, and fasting blood glucose of male apoE knockout mice, but total cholesterol tended to be higher in the HFD-PBS, the HFD-αGC, and the HFD-KO groups, compared to the SD-PBS, the SD-αGC, and the SD-KO groups (Table 1). Serum triglyceride and free fatty acid levels were significantly lower in the HFD-KO group than in the HFD-PBS group. Serum IFN-γ levels were significantly higher in the HFD-PBS group than in the SD-PBS group. In contrast, the elevated levels of serum IFN-γ in the HFD-PBS group was significantly ameliorated in the HFD-KO group (Table 1).

### 2.2. αGC-Induced iNKT Cell Accumulation in Aortic Tissues

**Figure 1:**
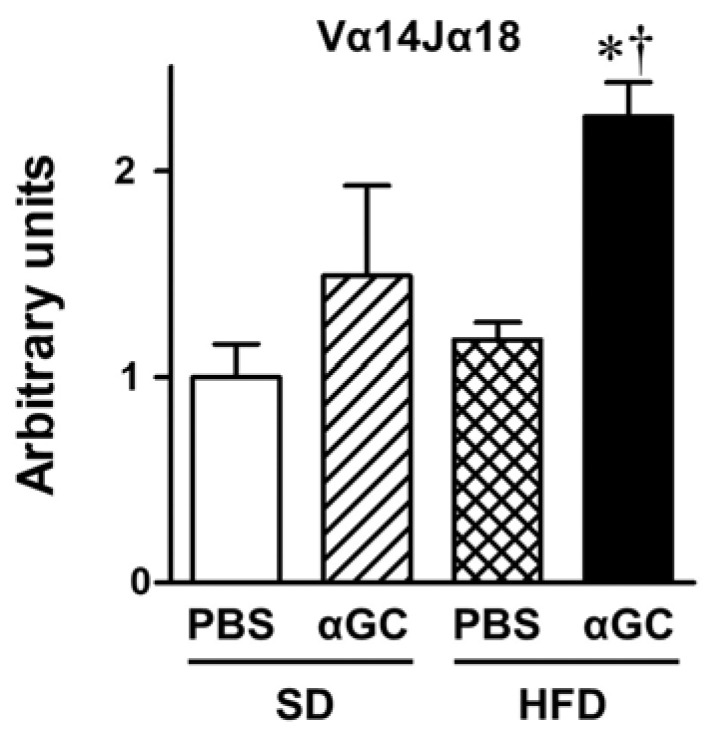
Gene expression of Vα14Jα18, a specific marker of iNKT cell accumulation, in aortic tissues from four groups of SD-PBS (n = 6), SD-αGC (n = 7), HFD-PBS (n = 21), and HFD-αGC (n = 21) mice. * *p* < 0.01 vs. SD-PBS, ^†^ *p* < 0.01 vs. HFD-PBS by ANOVA. All data are expressed as means ± S.E.

iNKT cell accumulation into aortic tissues quantified by Vα14/Jα18 gene expression was comparable between the SD-PBS and the HFD-PBS groups. αGC injection significantly enhanced iNKT cell accumulation in the HFD-αGC group, but not in the SD-αGC group (Figure 1).

### 2.3. Atherosclerotic Plaque Instability

**Figure 2:**
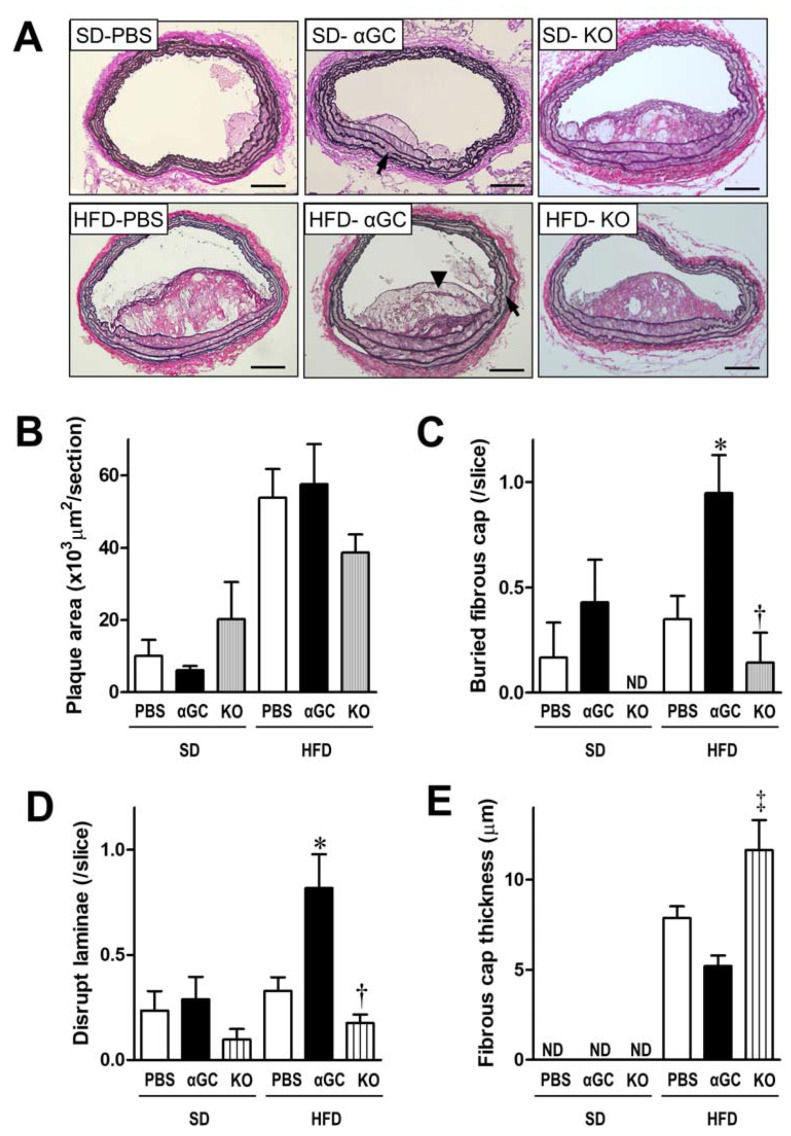
Histomorphometric analysis at the brachiocephalic artery from six groups of SD-PBS (n = 6), SD-αGC (n = 7), SD-KO (n = 3), HFD-PBS (n = 21), HFD-αGC (n = 21), and HFD-KO (n = 9) mice. (**A**) Representative photomicrographs of elastica-van Gieson’s staining of cross-sections. Scale bar = 100 μm. In HFD-PBS, HFD-αGC, and HFD-KO mice, arrows indicate disrupted elastic laminae and an arrowhead indicates buried fibrous cap. Summary data of plaque area (**B**), the number of buried fibrous caps (**C**), the number of disrupted elastic-laminae (**D**), and the maximal fibrous cap thickness (**E**) from six groups of mice. * *p* < 0.05 vs. HFD-PBS, ^†^ *p* < 0.05 vs. HFD-αGC, and ^‡^ *p* < 0.01 vs. HFD-αGC by ANOVA. All data are expressed as means ± S.E. ND, not determined.

Fatty streak lesions were observed in brachiocephalic artery from the SD-PBS group (Figure 2A and Figure S1A,B). αGC administration altered no structural changes in these SD mice. In contrast, complex fibro-atheromatous lesions with well-defined fibrous caps were present in the HFD-PBS group. Although acute plaque rupture, defined as a visible bleach in the cap with intraplaque hemorrhage, was not observed, the atherosclerotic plaque area appeared to be slightly higher in the HFD-PBS, the HFD-αGC, and the HFD-KO groups compared to the SD-PBS group (Figure 2A,B and Figure S1A,B). The number of buried fibrous caps, the signs of healed plaque ruptures, and the disrupted elastic laminae in the HFD-PBS group were nearly same as those in the SD-PBS group. However, the number of buried fibrous caps and disrupted elastic laminae, respectively, was significantly increased in the HFD-αGC group compared to the HFD-PBS group (Figure 2A,C,D). Fibrous cap thickness appeared to be slightly thinner in the HFD-αGC group than that in the HFD-PBS group (Figure 2E). In contrast, these increases in the number of buried fibrous caps and disrupted elastic laminae in the HFD-αGC group were completely ameliorated in the HFD-KO group (Figure 2A,C,D). Moreover, fibrous cap thickness appeared to be slightly greater in the HFD-KO group than that in the HFD-αGC group (Figure 2E). These findings suggest that the activation of iNKT cells by an αGC administration may enhance the instability of atherosclerotic plaque and depletion of iNKT cells may stabilize atherosclerotic plaque in HFD-fed mice.

### 2.4. Inflammation and MMP in Aortic Tissues

**Figure 3:**
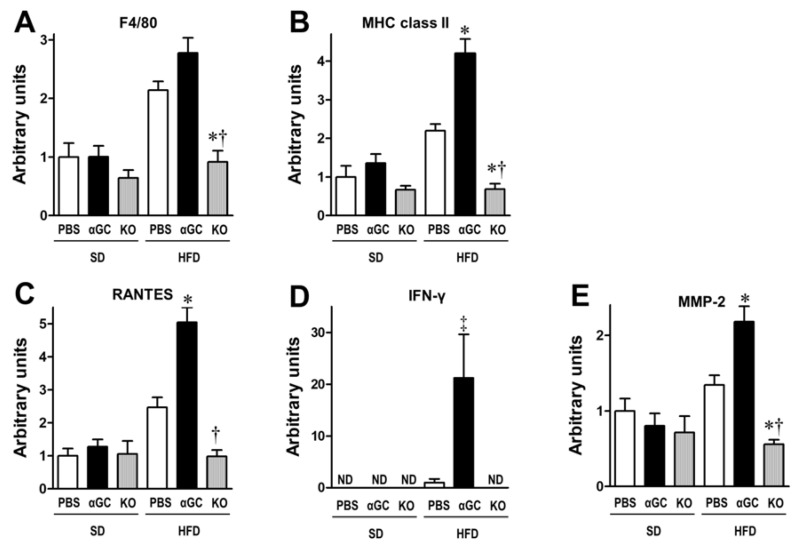
Gene expression of F4/80 (**A**), major histocompatibility complex (MHC) class II (**B**), regulated upon activation normal T cell expressed secretion (RANTES; (**C**)), interferon (IFN)-γ (**D**), and matrix metalloproteinase (MMP)-2 (**E**) in aortic tissues from six groups of SD-PBS (n = 6), SD-αGC (n = 7), SD-KO (n = 3), HFD-PBS (n = 21), HFD-αGC (n = 21), and HFD-KO (n = 9) mice. * *p* < 0.05 vs. HFD-PBS, ^†^ *p* < 0.01 vs. HFD-αGC by ANOVA, and ^‡^ *p* < 0.05 vs. HFD-PBS by student *t* test. All data are expressed as means ± S.E. ND, not detected.

F4/80 as well as MHC class II and regulated upon activation, normal T cell expressed and secreted (RANTES) gene expressions, markers of macrophage and T lymphocyte accumulation, respectively, appeared to be slightly greater in aortic tissues from the HFD-PBS group compared to those from the SD-PBS group. In contrast, an αGC administration enhanced the accumulation of inflammatory cells in the aortic tissues in the HFD-αGC mice (Figure 3A–C). In addition, αGC significantly increased IFN-γ gene expression in the HFD-αGC group (Figure 3D), suggesting that αGC enhanced the shift toward to Th1. In contrast, the depletion of iNKT cells in the HFD-KO group significantly attenuated the inflammatory cell’s accumulation and increased gene expression of IFN-γ observed in the HFD-αGC group (Figure 3A–D). Moreover, αGC increased MMP-2 gene expression, which enhances the matrix degradation within the aortic tissues, in the HFD-fed mice, and this increase was significantly attenuated in the HFD-KO mice where no iNKT cells were present (Figure 3E). An αGC administration showed no effect on the MMP-9 gene expression (data not shown).

**Figure 4:**
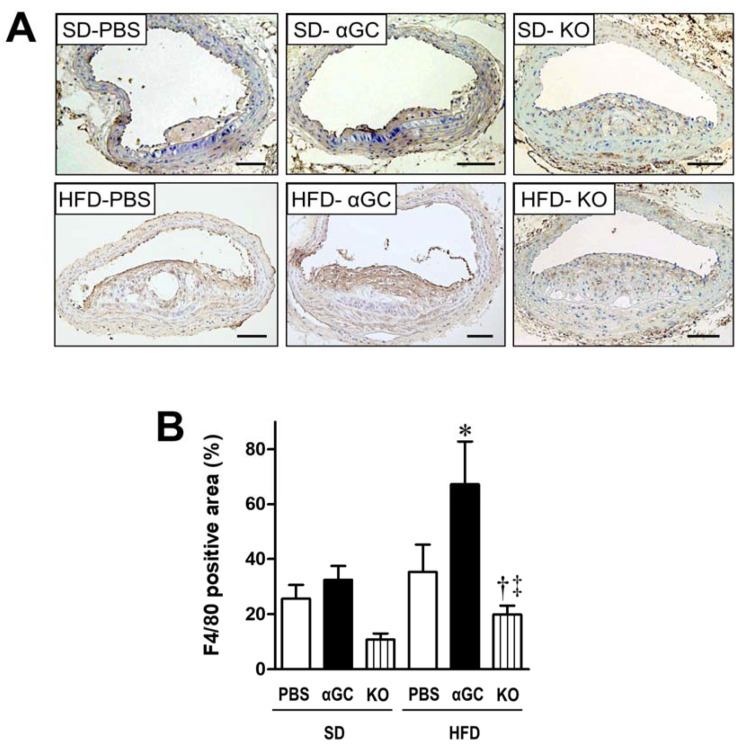
Representative photomicrographs of anti-F4/80 immunohistochemistry of cross-sections at the brachiocephalic artery (**A**) and summary data of anti-F4/80 positive area (**B**) from 6 groups of SD-PBS (n = 4), SD-αGC (n = 6), SD-KO (n = 3), HFD-PBS (n = 18), HFD-αGC (n = 16), and HFD-KO (n = 7) mice. Scale bar = 100 μm. * *p* < 0.01, ^†^ *p* < 0.05 vs. HFD-PBS, and ^‡^ *p* < 0.01 vs. HFD-αGC by ANOVA. All data are expressed as means ± S.E.

In parallel with increasing F4/80 gene expression, the infiltration of F4/80 positive macrophages by immunohistochemical staining was significantly increased in atherosclerotic plaques in HFD-αGC than HFD-PBS and this increase was significantly ameliorated in HFD-KO (Figure 4A,B). CD3-positive T lymphocytes were infiltrated into the atherosclerotic plaques in each group; however, their infiltration did not significantly differ among the groups (data not shown).

## 3. Discussion

The present study demonstrated that the activation of iNKT cells by an administration of αGC exacerbated atherosclerotic plaque instability via activating macrophages and T lymphocytes and upregulation of MMP-2 in a mouse model of plaque rupture. On the contrary, the depletion of iNKT cells in apoE/Jα18 double knockout mice significantly attenuated the inflammatory cell accumulation, upregulation of MMP-2 gene expression, and atherosclerotic plaque instability. These findings support the hypothesis that iNKT cells play a pivotal role in the pathophysiology of plaque instability by modulating inflammatory processes within the atherosclerotic wall.

Plaque rupture is a major cause of atherothrombotic events [2]. The infiltration and activation of macrophages and lymphocytes within the atherosclerotic lesion contribute to the plaque instability and subsequent plaque rupture [3]. iNKT cells are an innate-like T lymphocyte that recognize glycolipid antigens presented by the MHC class I-like molecule CD1d and is capable to rapidly and robustly produce a mixture of Th1 and Th2 cytokines, such as IFN-γ and IL-4, leading to subsequent immune responses on activation [4]. Thus, iNKT cells can function as a bridge between the innate and adaptive immune systems, and orchestrate tissue inflammation. We previously demonstrated that in vivo administration of αGC decreased collagen content and increased cellularity of atherosclerotic lesions in the aortic sinus from apoE knockout mice, suggesting that iNKT cells may affect the plaque instability [5]. However, the relevance of changes in the atherosclerotic lesions in the aortic sinus is rather limited because the incident plaque rupture is very rare in the aortic sinus [10,11]. In contrast, in an animal model that we employed in the present study, HFD feeding can develop advanced atherosclerotic lesions in the brachiocephalic artery from apoE knockout mice with several morphological features similar to human ruptured plaques [12,13]. Taking the preset findings and previous reports together, we confirmed that iNKT cells are involved in the arterial plaque instability.

The inflammatory cells accumulate within atherosclerotic plaques and produce pro-inflammatory cytokines as well as proteases, which may contribute to the plaque instability. Notably, IFN-γ, the primary Th1 cytokine secreted from T lymphocytes, has been reported to play an important role in plaque instability by inhibiting SMC proliferation and further reducing collagen synthesis within the vascular tissues [14]. In addition to SMC, immune cells from the fibrous cap of atherosclerotic lesions are sensitized to Fas-induced apoptosis by IFN-γ, which is one of the major contributing factors to the plaque rupture [15]. Moreover, the IFN-γ activates macrophages and upregulates the expressional levels of MMPs within atherosclerotic plaques, which can degrade collagens [14,16]. Activated T cells stimulate macrophages to produce MMPs via increased secretion of IFN-γ. In particular, MMP-2 and MMP-9 play an essential role in the pathogenesis of vascular remodeling. The present study demonstrated that iNKT cell activation by αGC increased MMP-2 expression and this increase was attenuated by the iNKT cell depletion (Figure 3E), whereas it did not affect MMP-9 gene expression within the aortic tissues. Activated macrophages secrete larger amounts of MMP-9 under the Th1 slant [17]. In contrast, IFN-γ has been shown to inhibit MMP-9 gene expression in macrophages [18]. Taken together, the increased IFN-γ might suppress the increase of MMP-9 by iNKT cell activation.

In addition, we demonstrated that activation of iNKT cells was associated with increased gene expression of F4/80, MHC class II, RANTES, IFN-γ, and MMP-2 in the aortic tissues (Figure 3A–E). Accordingly, cytokines, chemokines, and MMPs, including IFN-γ, were considered to be mechanistically involved in the plaque instability as a result of iNKT cell activation in our mouse model of spontaneously occurred plaque rupture. We previously demonstrated that macrophages conditioned with activated iNKT cells by αGC secreted greater amount of MCP-1 into the co-culture medium [19]. The iNKT cells may orchestrate the inflammatory process in association with the development of atherosclerotic plaque. Thus, iNKT cells appear to be involved in the enhancement of plaque instability via activating inflammatory cells in vascular tissues. We previously reported that iNKT cells accelerate atherogenesis and supportive reports have been accumulated [5,19]. Based on these findings, we speculate that iNKT cells may play a critical role from early to advanced stages of atherosclerosis.

Recent pathological analyses suggest that clinically critical plaques possess following each aspect; (i) numerous inflammatory cells, lipid-rich necrotic cores, and thin fibrous caps (plaque rupture) or (ii) abundant extracellular matrix and endothelial apoptosis (plaque erosion) [20]. The former aspect, plaque rupture, has been extensively studied and the underlying mechanism of necrotic core formation involves in the death of inflammatory cells (including macrophages), coupled with poor phagocytic clearance of these dead cells by a process called efferocytosis [21]. Macrophages are highly plastic cells and alter the efferocytotic function mediated by complex combinations of inflammatory cytokines, extracellular matrix, environmental factors such as hypoxia, and other inflammatory cells [22]. Further studies are needed for better understanding the inter- and intracellular impact on macrophage’s phenotype switch in the atherosclerotic lesions.

We previously showed that iNKT cells were infiltrated into visceral adipose tissues from HFD-induced obese mice in association with activation of macrophages [23]. Thus, the metabolic abnormalities may indirectly contribute to plaque instability in our model. Recent studies have demonstrated that obesity induces chronic inflammation in perivascular adipose tissues, suggesting direct relationships between metabolic derangements and vascular inflammation [24,25,26]. In our mouse model, iNKT cells may play a key role in chronic inflammation in both vascular tissues and adipose tissues, which may result in the development of plaque instability and glucose intolerance. However, we could not obtain sufficient amounts of perivascular adipose tissue around the brachiocephalic artery, and thus, we did not assess the link between inflammation in perivascular adipose tissues and plaque instability.

There are several limitations to be acknowledged in the present study. First, we could not directly demonstrate the distribution of iNKT cells in situ by the immunohistochemical analysis using CD1d dimer with loading of αGC, which specifically binds to Vα14/Jα18, according to a previous report [27]. We also tried to conduct the double immunohistochemical staining using antibodies for anti-TCR-β and anti-NK 1.1 according to the methods reported by another paper [28]. Furthermore, we performed in situ hybridization using DNA probes for mouse Vα14Jα18 as well as the flow cytometric analysis. Unfortunately, we could not directly show the distribution of iNKT cells in situ within the aortic tissues, although we could define iNKT cells and inflammatory cytokines by gene expression. Further studies are needed to overcome some technical difficulties of in situ detection and to demonstrate the distribution of iNKT cells in various types of lesions. Second, serum levels of triglyceride and free fatty acid were significantly decreased in the HFD-KO mice compared to the HFD-PBS mice. These alterations might have partly affected the plaque instability along with the attenuation of inflammatory gene expressions. iNKT cells may affect lipid metabolism; however, the causal relationship between them needs to be elucidated. Third, serum levels of IFN-γ were elevated in HFD-PBS and HFD-αGC; on the other hand, the expressional levels of IFN-γ in aortic tissues were significantly increased only in HFD-αGC group. The source of IFN-γ production after the stimulation of αGC remains to be determined. IFN-γ has been shown to be produced by iNKT cells themselves upon exogenous stimulation [4]. In addition, IFN-γ can be expressed and secreted from macrophages which are activated by iNKT cells. Alternatively, inflammatory cells in other tissues such as visceral adipose tissues may contribute to increase the serum levels of IFN-γ in the HFD-PBS mice. Molecular mechanisms that connect activating iNKT cells to priming immune response toward Th1 slant remains to be elucidated. Finally, the present study showed that administration of αGC increased gene expression of MMP-2 in the aortic tissue of the HFD-fed mice, but we could not show the change in the protein levels of MMP-2 in this tissue due to lack of samples.

In conclusion, the activation of iNKT cells by αGC exacerbated atherosclerotic plaque instability via activating macrophages and upregulation of MMP-2 in a mouse model of plaque rupture. On the other hand, the depletion of iNKT cells in apoE/Jα18 double knockout mice significantly attenuated the macrophage accumulation, upregulation of MMP-2 gene expression, and atherosclerotic plaque instability. Taken together, iNKT cells are involved in the exacerbation of atherosclerotic plaque instability via activating inflammatory cells and upregulation of MMP. The iNKT cells may be a novel therapeutic target against not only atherosclerosis but also plaque rupture.

## 4. Materials and Methods

### 4.1. Experimental Mice

Male apoE knockout mice (The Jackson Laboratory, Bar Harbor, ME, USA), 8 weeks of age, were fed an SD or HFD (containing 34.15% (*wt/wt*) sucrose, 21% (*wt/wt*) anhydrous milkfat, 19.5% (*wt/wt*) casein, 15% (*wt/wt*) corn starch, 5% (*wt/wt*) cellulose, 3.5% (*wt/wt*) mineral mix AIN-76, 1% (*wt/wt*) vitamin mix, 0.4% (*wt/wt*) calcium carbonate, 0.3% (*wt/wt*) DL-methionine, and 0.15% (*wt/wt*) cholesterol; Oriental Yeast Co. Ltd., Tokyo, Japan) for 8 weeks. The HFD-fed apoE knockout mice were divided into two groups according to the intraperitoneal injection of α-galactosylceramide (αGC 2 μg/mouse, Funakoshi Co. Ltd., Tokyo, Japan; HFD-αGC, n = 21) or phosphate-buffered saline (PBS; HFD-PBS, n = 21) twice a week for 8 weeks. SD-fed apoE knockout mice were also divided into two groups by the injection of αGC (SD-αGC, n = 7) or PBS (SD-PBS, n = 6).

Male apoE knockout mice and female Jα18 (previously defined as Jα281) knockout mice, which lack iNKT cells on the C57BL/6 background [29], were crossed to generate apoE/Jα18 double knockout mice. The apoE/Jα18 double knockout mice, 9 weeks of age, were also fed an SD or HFD for 8 weeks (SD-KO, n = 3 and HFD-KO, n = 9). 

At 16–17 weeks of age, these six groups of animals were intraperitoneally anesthetized with overdose of pentobarbital sodium (100 mg/kg) and euthanized by collection of blood from right ventricle, and organs, including the brachiocephalic artery and aortic tissues, were dissected via a thoracotomy. The animal care and procedures for the experiments (08-0267) were approved by the Committee of Hokkaido University Graduate School of Medicine on Animal Experimentation and conformed the Guide for the Care and Use of Laboratory Animals published by the US National Institutes of Health. 

### 4.2. Blood Chemistry

After fasting for 16 h, serum levels of total cholesterol, HDL-cholesterol, triglyceride, and free fatty acid were assayed by enzymatic methods (Wako Pure Chemical Industries, Ltd., Osaka, Japan). Serum IFN-γ levels were measured by enzyme-linked immunosorbent assay kit (R&D Systems, Inc., Minneapolis, MN, USA). Fasting blood glucose levels were measured by using an automatic blood glucose meter (Glutest Ace, Sanwa chemical, Nagoya, Japan).

### 4.3. Histomorphometric Analysis

Brachiocephalic arteries were fixed in 10% neutralized buffered formaldehyde and embedded in optimum cutting temperature compound (OCT; Sakura Finetek Japan Co., Ltd., Tokyo, Japan). Serial sections of 3 μm thickness at 30 μm intervals along the long axis of the artery were stained with hematoxylin and eosin, oil red O, or elastica-van Gieson. Atherosclerotic lesions were captured with a BZ-8000 microscope (Keyence Corp., Osaka, Japan) and analyzed using image analysis software (Image J version 1.43, National Institutes of Health, Bethesda, MD, USA).

Atherosclerotic plaque area was measured in oil red O-stained sections obtained throughout the brachiocephalic artery. Characteristics of ruptured atherosclerotic plaques in brachiocephalic arteries were assessed by the methods described by Jackson et al. [8]. Acute plaque rupture was defined as a visible bleach in the cap with intraplaque hemorrhage intruding into the lesion at the same site. Buried fibrous caps, defined as remnants of previous fibrous caps that have ruptured and been incorporated into the body of the plaque as it develops, was counted as indicative of healed plaque rupture on elastica-van Gieson-stained sections [30]. Disrupted elastic laminae within the body of the plaque were counted with serial sections at 30 μm intervals, and the mean number of disrupted elastic laminae per section was calculated throughout the artery. Maximal fibrous cap thickness was measured in the serial sections throughout the artery. 

To quantify the infiltration of macrophages and T lymphocytes to the atherosclerotic plaques in brachiocephalic arteries, three sections were stained with monoclonal antibody against mouse F4/80 (rat anti-mouse F4/80 monoclonal antibody, AbD Serotec, Kidlington, UK), a specific maker for mature macrophages, and mouse CD3 (hamster anti-mouse CD3 monoclonal antibody, AbD Serotec, Kidlington, UK), a specific maker for T lymphocytes, followed by counter-staining with hematoxylin. The degree of macrophage or T lymphocyte infiltration was expressed as a percentage based upon the ratio of the F4/80- or CD3-positive area to the total atherosclerotic plaque area.

### 4.4. Quantitative Reverse Transcriptase PCR

Total RNA was extracted from aortic tissues including distal aortic arch, left common carotid artery, and left subclavian artery with RNeasy mini kit (QIAGEN, Tokyo, Japan) according to the manufacturer’s protocol. cDNA was synthesized with the high-capacity cDNA reverse transcription kit (Applied Biosystems, Foster City, CA, USA). TaqMan quantitative PCR was performed with the 7300 real-time PCR system (Applied Biosystems) to amplify samples for Vα14/Jα18 (a specific marker of iNKT cells), F4/80, MHC class-II (a marker for macrophage activation), RANTES, IFN-γ, MMP-2, and MMP-9 cDNA. These transcripts were normalized to GAPDH. The sequences used to amplify Vα14/Jα18 are as follows: Forward; CTG GAG CAA CCA GAC AAG CTT, Reverse; GGT GGC GTT GGT CTC TTT GA, TaqMan Probe; CCT GCC AAG ATA TC. The other primers were purchased from Applied Biosystems.

### 4.5. Statistical Analysis

Data were expressed as the means ± S.E. Statistical analysis was performed using the ANOVA among six groups or student *t* test between HFD-PBS and HFD-αGC (GraphPad Prism 5, GraphPad Software, San Diego, CA, USA). A *p* value < 0.05 was considered statistically significant. If statistical significance was determined by the ANOVA, the data were further analyzed by the three series of Bonferroni post-hoc test to detect specific differences among the SD-fed groups, the HFD-fed groups, or between SD-PBS and HFD-PBS.

## Figures and Tables

**Table 1:** Animal characteristics.

	SD-PBS (n = 6)	SD-αGC (n = 7)	SD-KO (n = 3)	HFD-PBS (n = 21)	HFD-αGC (n = 21)	HFD-KO (n = 9)
Body weight, g	26.0 ± 0.7	25.8 ± 0.6	30.8 ± 1.6 *^†^	27.0 ± 0.5	26.0 ± 0.6	26.6 ± 0.5
Blood chemistry						
Total cholesterol, mg/dL	685 ± 19	708 ± 96	639 ± 47	1068 ± 65	1001 ± 103	961 ± 98
HDL cholesterol, mg/dL	15 ± 4	15 ± 2	28 ± 3	37 ± 8	34 ± 6	24 ± 5
Triglyceride, mg/dL	77 ± 5	84 ± 10	95 ± 7	221 ± 31	181 ± 27	84 ± 7 ^‡^
Free fatty acid, mEq/L	1.31 ± 0.13	1.18 ± 0.14	0.98 ± 0.14	1.37 ± 0.09	1.53 ± 0.08	0.93 ± 0.04 ^‡§^
Fasting blood glucose, mg/dL	67 ± 6	82 ± 13	89 ± 23	69 ± 3	65 ± 2	135 ± 11 ^‡§^
IFN-γ, pg/mL ^¶^	0.9 ± 0.4	1.3 ± 0.3	0.7 ± 0.2	29.4 ± 8.0 *	35.8 ± 7.7	0.5 ± 0.2 ^‡§^

PBS: phosphate buffered saline, αGC: α-galactosylceramide, KO: ApoE/J18 double knockout mice, SD: standard diet, HFD: high fat diet, HDL: high-density lipoprotein, IFN-γ: interferon gamma. * *p* < 0.05 vs. SD-PBS, ^†^ *p* < 0.05 vs. SD-αGC, ^‡^ *p* < 0.05 vs. HFD-PBS, and ^§^ *p* < 0.01 vs. HFD-αGC by ANOVA. ^¶^ IFN-γ was measured in the subgroups of the HFD-PBS (n = 7) and the HFD-αGC (n = 6) mice. All data are expressed as means ± S.E.

## Data Availability

The raw data supporting the conclusions of this article will be made available by the authors, without undue reservation.

## Supplementary Materials

The supplementary material is available online at https://www.mdpi.com/article/10.3390/ijms222212451/s1, Figure S1: Photomicrographs of brachiocephalic artery from four groups of SD-PBS (n = 6), SD-αGC (n = 7), HFD-PBS (n = 21), and HFD-αGC (n = 21) mice.

## Abbreviations

αGC	α-galactosylceramide
apoE	apolipoprotein E
HDL	high-density lipoprotein
HFD	high-fat diet
IFN-γ	interferon-γ
IL-4	interleukin-4
iNKT cell	invariant natural killer T cell
MHC	major histocompatibility complex
MMP	matrix metalloproteinase
NK cell	natural killer cell
RANTES	regulated upon activation, normal T cell expressed and secreted
SD	standard diet
SMC	smooth muscle cell
TCR	T cell receptor
Th1	T helper type 1
