# Supplementary material for: Natural Killer T Cells Are Involved in Atherosclerotic Plaque Instability in Apolipoprotein-E Knockout Mice
Source: Int J Mol Sci. 2021 Nov 18;22(22):12451. doi: 10.3390/ijms222212451 (PMC8618636; doi:10.3390/ijms222212451)
Supplement: Supplementary file 1 [file ijms-22-12451-s001.zip › ijms-1465540-supplementary.pdf]

Figure S1

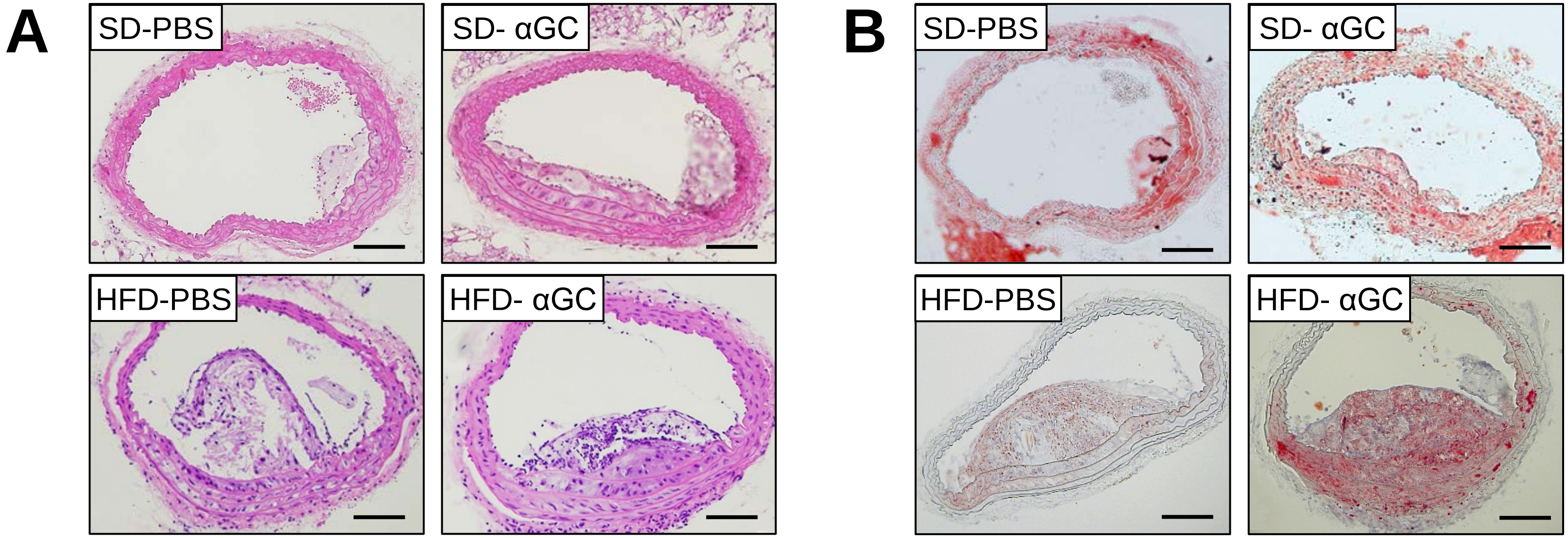

Figure S1. Photomicrographs of brachiocephalic artery from 4 groups of SD-PBS (n=6), SD-*α*GC (n=7), HFD-PBS (n=21), and HFD-*α*GC (n=21) mice. (A) Representative photomicrographs of Hematoxylin-Eosin staining of cross-sections. (B) Representative photomicrographs of Oil Red-O staining of cross-sections. Scale Bar=100μm.
